# Multi-tissue gene expression profiling of cows with a genetic predisposition for low and high milk urea levels

**DOI:** 10.1080/10495398.2024.2322542

**Published:** 2024-03-01

**Authors:** Henry Reyer, Hanne Honerlagen, Michael Oster, Siriluck Ponsuksili, Björn Kuhla, Klaus Wimmers

**Affiliations:** a Institute of Genome Biology, Research Institute for Farm Animal Biology (FBN), Dummerstorf, Germany; b Animal Breeding and Genomics, Wageningen University and Research, Wageningen, The Netherlands; c Institute of Nutritional Physiology ‘Oskar Kellner’, Research Institute for Farm Animal Biology (FBN), Dummerstorf, Germany; d Faculty of Agriculture and Environmental Sciences, Professorship of Animal Breeding and Genetics, University of Rostock, Rostock, Germany

**Keywords:** RNAseq, nitrogen emissions, mammary gland, liver, kidney

## Abstract

Milk urea (MU) concentration is proposed as an indicator trait for breeding toward reduced nitrogen (N) emissions and leaching in dairy. We selected 20 German Holstein cows based on MU breeding values, with 10 cows each having low (LMUg) and high (HMUg) MU genetic predisposition. Using RNA-seq, we characterized these cows to unravel molecular pathways governing post-absorptive body N pools focusing on renal filtration and reabsorption of nitrogenous compounds, hepatic urea formation and mammary gland N excretion. While we observed minor adjustments in cellular energy metabolism in different tissues associated with different MU levels, no transcriptional differences in liver ammonia detoxification were detected, despite significant differences in MU between the groups. Differential expression of *AQP3* and *SLC38A2* in the kidney provides evidence for higher urea concentration in the collecting duct of LMU cows than HMU cows. The mammary gland exhibited the most significant differences, particularly in tricarboxylic acid (TCA) cycle genes, amino acid transport, tRNA binding, and casein synthesis. These findings suggest that selecting for lower MU could lead to altered urinary urea (UU) handling and changes in milk protein synthesis. However, given the genetic variability in N metabolism components, the long-term effectiveness of MU-based selection in reducing N emissions remains uncertain.

## Introduction

In current agricultural systems, nitrogen (N) losses are of great importance due to their associated environmental burdens. These include the emission of ammonia and nitrous oxide into the atmosphere and the leaching of nitrate into ground water.^1^^,^^2^ Specifically for livestock farming, N losses arise from the uncontrolled input of ammonia, nitrous oxide, di-N and nitrate, which mainly originates from stable or free-range farming and from the storage and spreading of manure.

Ruminants, such as dairy cows have the unique ability to recycle urea N *via* the microbial community in the rumen. Nevertheless, they exhibit low N use efficiencies in current farming systems, with approximately 70% of daily N intake being excreted in urine and feces.^1^ Including a certain proportion of N for the basic metabolic rate, on average only 25% of dietary N supplied with the crude protein is converted into milk, reflecting a low N utilization efficiency (NUE).^3^^,^^4^ This is due to the fact that the inclusion of a high dietary crude protein content is favorable for higher milk production rates, which however decreases the NUE.^5^ When dietary N intake is lower, the proportion of urea N recycled in the rumen-intestinal tract is usually higher.^6^ Rumen microbes utilize urea N to build additional microbial protein, which is a highly valuable amino acid source for the anabolic purposes of the cow.^7^ Individual variation in the digested N fraction recycled by microbes was reported to range between 4 and 73%.^8^

The physiological routes involved in urea N recycling include the hepatic conversion of ammonia originating from the ruminal lumen, from the hepatic degradation of amino acids or from extrahepatic organs entering the liver *via* the bloodstream. The resulting urea, which in turn is released into the bloodstream, can subsequently enter the rumen *via* the rumen epithelia and saliva. In addition, a proportion of blood urea is released actively and passively through the mammary gland into the milk (milk urea [MU]) and *via* the kidney into urine (urinary urea [UU]). Given the relationships between blood urea, MU and UU^9^^,^^10^ and the fact that MU is routinely determined as part of milk performance testing to monitor the N feeding supply, MU has recently been proposed as a predictor of N excretion in the breeding of cows.^11^^,^^12^ Estimates of heritability for MU across different lactation stages and lactation numbers revealed values ranging from about 0.13 to 0.59, whereas correlations with other milk traits are low, suggesting a potential for MU as a suitable breeding trait.^13^^,^^14^

For this study, a breeding value for MU was estimated based on milk records of about 8 million Holstein lactations.^14^^,^^15^ This was used to assess the genetic predisposition of cows for MU concentration and to group the cow population of this study for high and low MU breeding values. Previous studies on rumen samples from these cows and others revealed shifts in the abundances of specific microbial taxa and rumen epithelial expression profiles, suggesting immune-related features that might modulate the interaction between host and microbiota.^16^^,^^17^ From a biological perspective, the observed ruminal differences between cows with contrasting breeding values for MU might be accompanied by differences in host gene expression in liver, kidney and mammary gland, which contribute as key tissues to the partitioning of N in the body pool in lactating ruminants. Accordingly, this study was designed to explore the consequences of a potential selection with MU at the level of post-absorptive tissues by means of transcriptomic profiling. A total of 20 Holstein cows with higher or lower breeding values for MU (HMUg and LMUg) were investigated to elucidate the affected molecular pathways.

## Material and methods

### Ethical statement

The animals were kept and samples were taken in accordance with the guidelines of the German Animal Protection Law, with the corresponding protocols approved by the ethical review board of the Research Institute for Farm Animal Biology (FBN). The research was done under the authority of the state office of Agriculture, Food Security and Fishery Mecklenburg-Western Pomerania, Rostock, Germany (LALLF M-V/7221.3-1-052/17).

### Animal trial and sample collection

Twenty lactating, multiparous Holstein cows divergent in breeding values for MU were kept and monitored in housing facilities of the FBN Dummerstorf over a two weeks adaptation period after acquisition from commercial farms. Details about housing conditions, animal handling and feeding were described for the same cows previously.^18^ The experimental trial lasted for two weeks and was carried out in 10 blocks. In each block, one HMUg and one LMUg cow were examined simultaneously and dry matter intake was recorded. HMUg and LMUg were determined by high and low breeding values for MU according to the estimates of the official breeding evaluation center for livestock in Germany (VIT, Verden, Germany).^14^^,^^15^ The estimated breeding values for MU were 36.8 ± 6.8 (unitless; mean ± SD) for HMUg cows and −22.6 ± 12.8 for LMUg cows. Phenotypic MU concentrations of the five latest milk records before the trial were HMUg = 278.1 ± 15.8 mg/L and LMUg = 181.54 ± 15.08 mg/L (mean ± SD), with comparable milk yields between the groups. For tissue sampling, the cows were stunned with a captive bolt and slaughtered by exsanguination in the morning four hours after milking and feeding. At slaughter, tissue samples from the liver (*lobus caudatus*), kidney (represented by cortex and medulla), and mammary gland (parenchymal tissue) were obtained. The samples were quickly prepared, frozen in liquid N and kept at −80 °C until RNA isolation.

### RNA extraction and sequencing

For RNA isolation, liver, kidney and mammary gland samples were ground to powder in liquid N and homogenized in TRI reagent (Sigma-Aldrich, Taufkirchen, Germany) using a syringe and needle, followed by extraction of the RNA according to the TRI reagent protocol. Thereafter, DNaseI digestion (Roche Diagnostics, Mannheim, Germany) and RNA purification with the NucleoSpin RNA extraction kit (Macherey-Nagel, Düren, Germany) were performed. The resulting RNA was checked on a 2100 Bioanalyzer instrument (Agilent Technologies, Santa Clara, CA). Across all tissues, the average RNA integrity number (RIN) was 7.6. Sequencing libraries were generated with sample-specific indices using the TruSeq Stranded mRNA kit (Illumina, San Diego, CA). Prior to sequencing on an Illumina HiSeq 2500 (Illumina), quality and quantity of generated libraries were checked with an Agilent DNA-1000 chip (Agilent Technologies) and the Qubit 3.0 fluorometer (Life Technologies, Darmstadt, Germany), respectively. The libraries were sequenced paired-end with 2 × 71 cycles.

### Data analysis

Sequencing reads were quality filtered (reads with mean Q-score < 20 and short reads < 20 bp) and trimmed (adapter sequences at the 3′ end) using Trim Galore version 0.6.5 (RRID:SCR_011847).^19^ Afterwards, pair-end reads were aligned to the Bos_taurus.ARS-UCD1.2 reference genome (Ensembl release 100, accessed on September 2020) with Hisat2 version 2.2.0 (RRID:SCR_015530).^20^

The initial quality check for gene expression data included the distance between individual data sets, signal intensity distribution and individual data set quality visualized using the arrayQualityMetrics R package. Accordingly, expression data of two kidney samples from the same experimental block (one HMUg and one LMUg cow) were excluded from further analysis. Differentially expressed genes (DEG) were identified using the Wald test implemented in the DESeq2 R package. Very low abundant transcripts were initially filtered, keeping only transcripts with at least 100 sequencing counts in at least five samples. The statistical model was designed to test the effect of cows with divergent breeding values for MU, while controlling for the effects of the experimental block, the individual number of lactations and the dry matter intake during the trial period. Human orthologous gene identifiers (gene symbols) were obtained from the Ensembl database based on the cattle-specific Ensembl identifiers using g:profiler (https://biit.cs.ut.ee/gprofiler). Genes with a *q* value < 0.05 in the comparison of HMUg and LMUg groups were considered as DEGs. Based on the rlog-transformed expression data from all three tissues, a multivariate analysis was performed using the DIABLO framework implemented in the R package mixOmics to integrate all molecular changes in the divergent cows. After estimating tuning parameters to determine the number of features in each of the datasets, 100, 25 and 25 genes were considered for the mammary gland, liver and kidney, respectively, to discriminate the HMUg and LMUg groups. The lists of DEGs and selected features were used for canonical pathway analysis with the Ingenuity Pathway Analysis tool (IPA; Qiagen, Hilden, Germany) and for the enrichment analysis in biological process (BP) terms of the Gene Ontology (GO) considering term sizes from 5 to 250 with g:profiler. Terms and pathways with a Benjamini–Hochberg adjusted *p* value < 0.05 were classified as significantly enriched.

In addition, functional candidate genes involved in liver, kidney, and mammary gland N metabolism were selected and evaluated in the dataset. Specifically, genes involved in amino acid transport,^21^ tricarboxylic acid (TCA) cycle (GO:0006099), urea cycle,^22^ and urea transport (GO:0015840) were investigated for their tissue-specific fold-change in expression values between HMUg and LMUg, considering a *p* value < 0.05 to indicate nominal significance.

## Results

The data obtained from RNA sequencing comprised 22.1 ± 2.4 million reads (mean ± SD) per sample across all three tissues and had an overall alignment rate to the reference of 98.6%. Transcriptomic analysis of liver, kidney and mammary gland samples revealed 84, 11 and 165 DEGs, respectively, comparing HMUg and LMUg cows (*q* < 0.05). The full lists of genes and the statistical evaluation are shown in Supplementary Table 1. The analysis of GO-terms and KEGG pathways based on the DEGs in liver and kidney did not reveal any enrichment. In the mammary gland, significantly enriched GO terms of BPs mainly comprised cellular amino acid metabolic processes, including those involving the transfer of amino acids to tRNAs (Table 1). For KEGG pathways, the extracellular matrix (ECM)-receptor interaction, focal adhesion, aminoacyl-tRNA biosynthesis and cholesterol metabolism were significantly enriched considering the DEGs identified in the mammary gland.

**Table 1.** Enrichment analysis based on genes differentially expressed in the mammary gland between cows with high and low breeding values for milk urea.

Database	Term name	Adjusted *p* value	Genes
GO:BP	cellular amino acid metabolic process	0.0004	*ASNS*, *DBT*, *DHFR*, *EPRS1*, *ETFA*, *GARS1*, *IARS1*, *MARS1*, *NARS1*, *PSAT1*, *TARS1*
GO:BP	tRNA aminoacylation for protein translation	0.0007	*EPRS1*, *GARS1*, *IARS1*, *MARS1*, *NARS1*, *TARS1*
GO:BP	tRNA aminoacylation	0.0011	*EPRS1*, *GARS1*, *IARS1*, *MARS1*, *NARS1*, *TARS1*
GO:BP	amino acid activation	0.0012	*EPRS1*, *GARS1*, *IARS1*, *MARS1*, *NARS1*, *TARS1*
KEGG	ECM-receptor interaction	0.0000	*CD36*, *COL4A2*, *COL6A3*, *HSPG2*, *ITGA5*, *ITGB4*, *LAMB2*, *THBS4*, *VWF*
KEGG	focal adhesion	0.0006	*COL4A2*, *COL6A3*, *ITGA5*, *ITGB4*, *LAMB2*, *PDGFRB*, *PIP5K1B*, *THBS4*, *TLN1*, *VEGFA*, *VWF*
KEGG	aminoacyl-tRNA biosynthesis	0.0006	*EPRS1*, *GARS1*, *IARS1*, *MARS1*, *NARS1*, *TARS1*
KEGG	cholesterol metabolism	0.0143	*CD36*, *LRP1*, *PLTP*, *SCARB1*, *TSPO*

**Figure 1.**
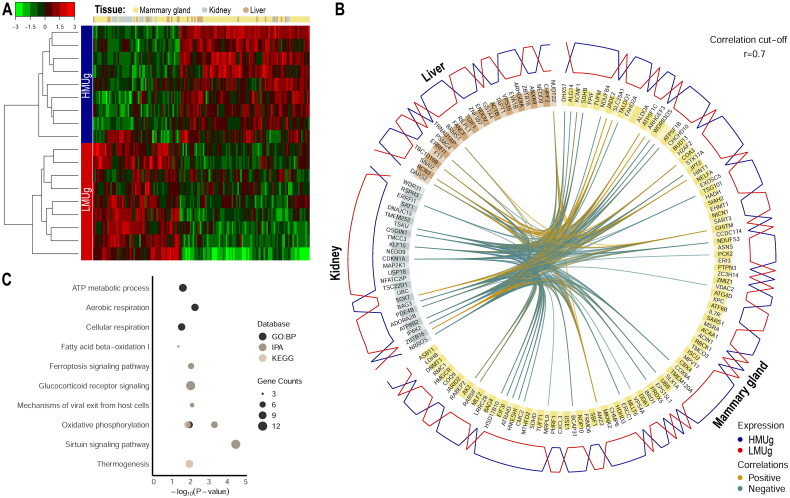
Expression profiling and enrichment analysis of key molecular features in liver, kidney and mammary gland discriminating HMUg and LMUg cows. The tissue-specific expression of the selected genes and the hierarchical clustering of the samples are illustrated in a heatmap (A). The color key represents the Z-score indicating the level of gene expression. The circos plot (B) indicates the key genes in each tissue (yellow: mammary gland, brown: liver and grey: kidney), their correlations greater than 0.7 (inner circle) and the predominant expression of genes in HMUg and LMUg group (outer circle). The enrichment plot (C) shows the significantly enriched GO terms (GO:BP) and pathways (IPA and KEGG) based on the 147 genes.

The holistic expression profiles were further integrated with DIABLO to determine key molecular factors in the three tissues that allowed a clear differentiation between HMUg and LMUg groups (Fig. 1). The 150 selected genes (100 from mammary gland, 25 from kidney and 25 from liver) comprised a set of 147 unique gene identifiers that were subjected to further analysis of enriched pathways and GO terms. The DEGs were found to be enriched in oxidative phosphorylation, ATP metabolism, thermogenesis as well as in IPA canonical pathways of Sirtuin Signaling, Glucocorticoid Receptor Signaling and Fatty Acid β-oxidation I (Fig. 1C).

**Figure 2.**
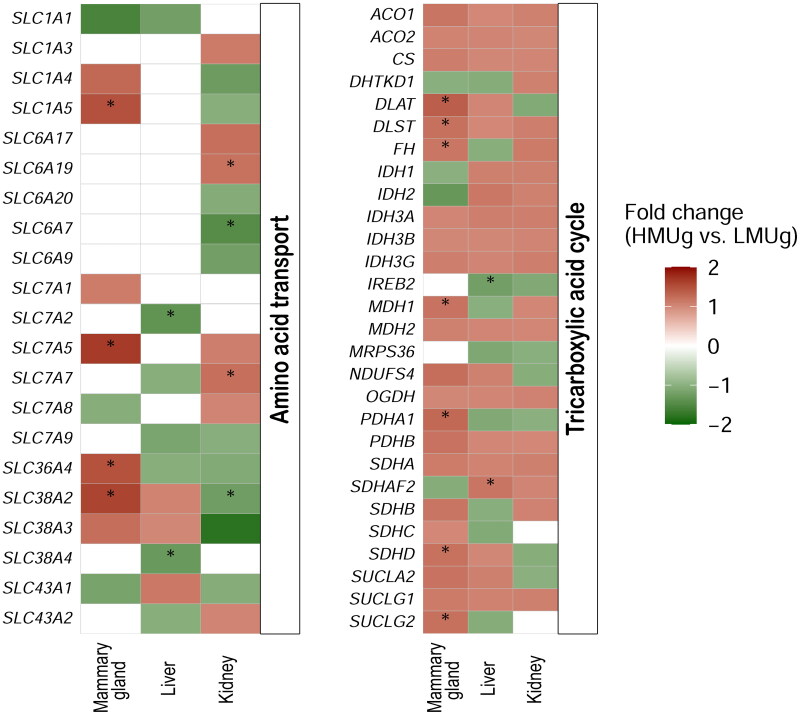
Genes related to amino acid transport and the TCA cycle and their differential expression in the mammary gland, liver and kidney between cows with high and low breeding values for MU. The colors reflect the fold change in expression levels between HMUg and LMUg cows. Asterisks indicate the significance of the results (*p* value < 0.05). White fields are indicating the absence of expression in the corresponding context.

The expression of genes in the themes amino acid transport, TCA cycle, urea cycle and urea transport was analysed in order to take a closer look at the most important genes known to be involved in N-metabolism in the three tissues. The amino acid transporters *SLC1A5*, *SLC7A5*, *SLC36A4* and *SLC38A2* for the transport of neutral amino acids were more abundant in the mammary gland of HMUg compared to LMUg (Fig. 2). *SLC7A2* and *SLC38A4*, transporters for cationic and neutral amino acids, respectively, were identified as DEG in the liver, with lower abundance in HMUg compared to LMUg. In the kidney, *SLC6A7* (proline transporter) and *SLC38A2* (transport of neutral amino acids) were significantly more abundant in HMUg than in LMUg cows, while *SLC6A19* (transport of neutral amino acids) and *SLC7A7* (transport of cationic and neutral amino acids) were less abundant in HMUg. For the TCA cycle, a number of genes were found to be significantly higher expressed in the mammary gland of HMUg compared to LMUg (Fig. 2). The gene panel representing urea transport included *UPK3A*, *SLC14A1*, *SLC14A2*, *AQP3* and *AQP7*, with only aquaporin 3, encoded by *AQP3*, being differentially expressed in the kidney (HMUg < LMUg). None of the genes involved in the urea cycle (*ARG1*, *ASL*, *ASS1*, *CPS1*, *NAGS*, *OTC*, *SLC25A15* and *SLC25A13*) were found to be significantly differentially expressed in liver and mammary gland. In kidney, *ASS1*, which encodes argininosuccinate synthase 1, was more abundant in HMUg compared to LMUg.

## Discussion

MU levels are routinely recorded as part of the monthly milk monitoring in dairy cows. The resulting phenotype availability and the physiological role of urea as a molecule of N excretion and N recycling at the same time, promoted MU as a breeding trait to reduce N excretion in ruminants.^13^^,^^23^^,^^24^ These circumstances were exploited to estimate a genomic breeding value for MU,^14^^,^^15^ which provided the basis for an in-depth investigation of animals with a genetic predisposition for high and low MU in this study. Considering the metabolism of N in ruminants, the focus of this research was on gene expression profiles in the liver, kidney and mammary gland, in addition to the mechanisms already studied in the rumen^15^ or in relation to nutritional aspects.^18^^,^^25^^,^^26^

Ammonia absorbed from the rumen is detoxified by the cow’s liver *via* the urea cycle. In addition to detoxifying ruminal ammonia, the liver also plays a key role in the catabolism of endogenous amino acids forming urea. This study revealed only a limited number of significant DEGs in the liver when comparing expression profiles of HMUg and LMUg cows. The subsequent data analysis revealed no significantly enriched GO terms and pathways, nor did the urea cycle genes appear to be differentially expressed. The latter is in line with previous results showing no differences in the expression of urea cycle or N-metabolism-associated genes at the level of mRNA and protein abundance in liver between cows with different MU concentrations.^27^ Two aspects could be of importance, first that cows with low and high MU concentrations have no significant differences in ruminal fluid ammonia concentrations,^18^ and second that the cow’s liver generally has a high capacity to detoxify ammonia, which would not require an extra effort of transcriptional adaptation at the urea cycle level.^28^ However, it is also conceivable that the genetic predisposition for MU directly acts on urea cycle capacity *via* polymorphisms within the urea cycle enzymes or in upstream regulators, such as *SIRT3.*^29^^,^^30^ Among the most prominent hepatic DEGs of this study some are involved in liver regeneration and metabolism (*NR1I2*, *SLC25A47* and *CRELD2*) as well as in the regulation of glycogen stores (*PPP1R3C*), suggesting subtle changes in liver metabolism between HMUg and LMUg animals.^31–33^ Interestingly, disruption of hepatic glucagon signaling in mice has been shown to reduce the expression of the amino acid transporters *SLC38A4* and *SLC7A2*, which were also differentially expressed between HMUg and LMUg in this study, thus impairing the availability of amino acids for gluconeogenesis.^34^

The kidneys absorb urea from the bloodstream and are responsible for its concentration and excretion in the urine. In addition, renal ammonia excretion can also have a variable contribution to the urinary N balance.^35^ Renal urea absorption along the nephron is mainly regulated by vasopressin and involves both, the activity of UT-A urea transporters encoded by *SLC14A1* and the abundance of aquaporins (e.g. AQP3 and AQP7) in the plasma membrane of the inner medullary collecting duct.^35^^,^^36^ The present analysis of renal gene expression revealed a numerically higher abundance of *SLC14A1* (FC = 2.58, *p* = 0.114, HMUg < LMUg) and a significantly higher abundance of *AQP3* (FC = 1.30, *p* = 0.003, HMUg < LMUg) in LMUg compared to HMUg, which might indicate an increased urea reabsorption into the interstitium in LMUg. In accordance, *SLC38A2* (FC = 1.24, *p* = 0.029, HMUg < LMUg), which has recently been shown to protect the renal medulla under hyperosmotic concentrations during urine concentration,^37^ was also found to be higher abundant in LMUg compared to HMUg. This could indicate for a compensatory transcriptional adaptation to the higher urea concentration in the medulla and, overall for an increased concentration of urea in the collecting duct of LMUg compared to HMUg animals. Interestingly, cows classified as having lower MU levels revealed a 1.4-fold higher UU N to MU N excretion rate compared to HMU cows.^18^ However, the absolute UU excretion remained unchanged between HMU and LMU cows.^18^ With regard to renal ammonia processing, only a difference in expression of sodium-dependent neutral amino acid transporter B(0)AT1, encoded by *SLC6A19*, was detected (FC = 1.20, *p* = 0.028, HMUg > LMUg), which might indicate a reduced transport of glutamine from the lumen as substrate for ammoniagenesis in LMUg compared to HMUg cows. However, the holistic analysis of gene expression profiles in the kidney revealed only a small number of DEGs and the subsequent integration analysis was insufficient to detect significantly enriched GO terms. Considering genetics, previous studies have indicated polymorphic genomic regions and candidate genes that may influence urea concentration in cows,^38^^,^^39^ as well as polymorphisms in human genes involved in ammonia metabolism that have been shown to influence ammoniagenesis and urinary pH.^40^

The N metabolism in the mammary gland is of great importance for milk production. This primarily involves the utilization of amino acids for incorporation into milk protein, as a source of energy, or as a carbon source for the synthesis of other, non-essential amino acids.^8^ Urea enters the milk by diffusion from the blood, thus the urea concentration in the milk largely corresponds to the urea concentration in the blood. In accordance to the selection of the examined cows on their breeding value for MU, the LMUg cows showed a lower MU concentration than the HMUg cows.^15^ Based on the RNAseq data, genes encoding aminoacyl-tRNAs, which catalyse the ligation of amino acids to their respective transfer RNAs, were found to be enriched. The identified tRNA synthetases perform the transfer of glutamate/proline (*EPRS1*), glycine (*GARS1*), isoleucine (*IARS1*), methionine (*MARS1*), asparagine (*NARS1*) and threonine (*TARS1*) and contribute to protein synthesis. Notably, all these genes were found to be higher abundant in HMUg compared to LMUg cows. Likewise, amino acid transporters encoded by *SLC1A5*, *SLC7A5*, *SLC36A4* and *SLC38A2* were higher abundant in HMUg compared to LMUg, facilitating the uptake of amino acids from the blood into the mammary gland and affecting the available capacity for protein synthesis.^41^ In this context, casein synthesis genes were also found to be more abundant in HMUg compared to LMUg (*CSN1S1* (FC = 1.57, *p* = 0.036), *CSN1S2* (FC = 1.63, *p* = 0.021) and *CSN3* (FC = 1.57, *p* = 0.027)). So far, a weak positive genetic correlation between MU and milk protein percentage has been described in Holstein cows,^14^ but no difference in milk protein was found between HMUg (3.64 ± 0.41%) and LMUg (3.72 ± 0.34%) cows in this study. However, positive correlations between beta-casein fractions and MU (0.31) and negative correlations between kappa-casein (−0.20) and beta-lactoglobulin (−0.26) fractions and MU have recently been estimated.^42^ Interestingly, the expression of milk proteins, especially beta-casein, has been described to be regulated by the ECM *in vitro,*^43^^,^^44^ which was also one of the enriched signaling pathways in the current mammary gland analysis. Whether the potentially different amino acid absorption and ligation between groups affect milk protein composition remains to be investigated in future studies. Protein synthesis in the mammary gland is an energy-intensive process, which might explain the parallel differences in gene expression of the components of the TCA cycle.^45^ According to the other observations, certain genes of the TCA cycle were consistently higher abundant in HMUg compared to LMUg. It seems that HMUg cows potentially take up more amino acids from the circulation and utilize these amino acids in an anaplerotic fashion in the TCA cycle for energy production. The energy might be used for milk fat synthesis, because HMUg cows have a higher milk fat concentration than LMUg cows.^18^ However, the fate of the remaining N following the utilization of amino acids in the TCA cycle remains unclear, not at least because no differences in the expression of urea cycle genes in the mammary gland have been observed.

The integration of gene expression data from the three tissues in a supervised analysis in order to identify factors discriminating between cows with a genetic predisposition for high and low MU resulted in a set of 147 transcripts. Based on the functional annotation of these genes, the enrichment predominantly represents features of cellular energy metabolism. Adequate energy supply is a central aspect of the metabolism of high-performing Holstein cows, and it has been shown that breeding selection for specific milk production traits indeed modulates energy metabolism pathways of dairy cows.^46^ Another aspect not represented in the tissue panel examined in this study is the transport of ammonia and urea in the blood facilitated by red blood cells, which involve aquaporins, urea transporter B (UT-B, SLC14A1) and the Rh-associated glycoprotein (RHAG).^47^^,^^48^ For mammalian species, polymorphisms in the coding sequence of the corresponding genes have been described, which putatively influence their transport activity and selectivity.^49^^,^^50^

## Conclusion

The transcriptome analysis comprised the comparison of cows with a genetic predisposition for high and low MU excretion with a focus on liver, kidney and mammary gland. In liver, no conclusive alterations of gene expression related to urea cycle were identified, suggesting that the pathways to detoxify ammonia are effective. At the kidney level, evidence for alterations in the expression of key genes for urea processing was observed, indicating an increased concentration of urea in the collecting ducts of LMUg compared to HMUg animals. Pronounced changes in gene expression profiles were observed in the mammary gland. Here, consistent findings implicate an effect on the synthesis of milk proteins, which demands further investigation into the consequences for milk protein quality. In all tissues, the selection of cows for MU breeding values was accompanied by subtle changes at the level of energy metabolism. The fact that urea concentration in milk is considered a generally suitable indicator of UU excretion at herd level does not seem to apply at individual animal level. The transcriptome analysis rather supports the assumption that the breeding for low MU could merely cause the partitioning of N-fractions toward urine. This, in turn, may contribute to a shift in the distribution of N in other compartments. Whether this results in quantitatively higher or lower N emissions cannot be deduced from the results of this study. However, several polymorphisms in genes of the N-metabolism are described in mammalian species that offer the potential to select for low N emission using phenotypes closer to the target variable.

## Supplementary Material

Supplemental Material

## Data Availability

The data that support the findings of this study are available from the corresponding author [KW] upon reasonable request.
